# A study of high neuroticism in long-term survivors of childhood, adolescence, and young adult cancers

**DOI:** 10.1038/s41598-022-15697-3

**Published:** 2022-07-19

**Authors:** Alv A. Dahl, Cecilie Essholt Kiserud, Sophie D. Fosså, Jon Håvard Loge, Kristin Valborg Reinertsen, Ellen Ruud, Hanne C. Lie

**Affiliations:** 1 National Resource Center for Late Effects After Cancer Treatment, Oslo University Hospital, Radiumhospitalet, Nydalen, P.O. Box 4953, 0424 Oslo, Norway; 2 Department of Behavioural Medicine, Institute of Basic Medical Sciences, University of Oslo, 0316 Oslo, Norway; 3 Department of Oncology, Oslo University Hospital, 0406 Oslo, Norway; 4 Faculty of Medicine, University of Oslo, 0316 Oslo, Norway; 5 Department of Paediatric Medicine, Oslo University Hospital, Rikshospitalet, 0029 Oslo, Norway

**Keywords:** Cancer, Psychology, Medical research, Oncology, Signs and symptoms

## Abstract

Neuroticism is a basic personality trait concerning negative feelings under stressful conditions. Our purpose was to examine the rate of high neuroticism and factors associated with high neuroticism in long-term (≥ 5 years) survivors of childhood, adolescent, and young adult cancer (CAYACSs). Norwegian CAYACSs aged 0–39 years when diagnosed and treated between 1985 and 2009 for cancer in childhood/adolescence (0–18 years), or as young adults (19–39 years) and alive in 2015 were mailed a questionnaire. Data from 1629 CAYACSs (481 children/adolescents and 1148 young adults) were analyzed. High neuroticism was found in 44% of survivors of childhood/adolescent cancers versus 34% in survivors of young adult cancer (p < 0.001). The rate of high neuroticism in female CAYACSs was 40% and in males 30% (p < 0.001). The corresponding difference between male survivor group was non-significant. In multivariable analysis, young age at survey, more adverse effects, poor self-rated health, female sex, chronic fatigue, and increased depression remained significantly associated with high neuroticism. Cancer treatment, comorbidity, and lifestyle were significant in bivariate analyses. Cancer at earlier age could increase the risk of high neuroticism among adult survivors. Screening for neuroticism could identify CAYACSs at risk for experiencing multiple health concerns and needing special follow-up attention.

## Introduction

Since the mid-1970s psychosocial concepts like quality of life, mental distress, and tiredness (fatigue) in cancer survivors have become familiar to the oncological community through numerous research papers^1^. Less research has focused on the relevance of personality and basic personality traits for survivorship problems. Among such traits, neuroticism is particularly relevant since it is strongly associated with, the abovementioned concepts. Neuroticism, like other basic personality traits, are determined by heredity and environment, and they are firmly established at young adult age. Thereafter, such traits tend to remain stable, but somewhat changing and modified during the rest of the life span^2,3^.

Neuroticism is the propensity to experience negative emotions, including anxiety, fear, sadness, anger, guilt, disgust, irritability, loneliness, worry, self-consciousness, dissatisfaction, hostility, embarrassment, reduced self-confidence, and feelings of vulnerability, in reaction to various types of stress”^4^. In general, high levels of neuroticism increase the risk of an unhealthy lifestyle, many somatic diseases, mental disorders, and premature death in general, but not of cancer-related death in particular^5–7^. However, studies also have indicated that persons with high neuroticism take better care of their health, particularly if they are anxious or worried^8^ or quite conscientious (healthy neuroticism)^9^.

Available literature databases hardly contain studies of neuroticism in long-term (≥ 5 years) survivors of childhood and adolescence cancers (aged 0–18 years at first cancer diagnosis) (CACSs) or young adult cancers (aged 19–39 years at first cancer diagnosis) (YACSs). Due to eventual negative health consequences, studies of high neuroticism among such long-term cancer survivors are of considerable clinical relevance.

Early childhood personality components like urgency, negative affect, and effortful control have been studied longitudinally in small samples of childhood cancer survivors. The conclusion was that these components were strong predictors of later psychosocial functioning^10^. Diagnosis and treatment of cancer could represent a trauma that affects later personality development^11^. Since the cancer trauma strikes earlier in the development of CACSs than of YACSs, there could be a risk of increase in high neuroticism among CACs compared to YACSs^12^.

In 2015/2016, a population-based cross-sectional health survey was performed among Norwegian CACSs and YACSs (CAYACSs when taken together) (The NOR-CAYACS study)^13^. By mailed questionnaires CAYACSs were invited to provide cancer-related, socio-demographic, health-related, and lifestyle information and to rate their level of neuroticism. Neuroticism was self-reported using the same scale as in the population-based Norwegian HUNT-3 health study (described later). Accordingly, we defined four research aims: (1) To investigate the occurrence of high neuroticism among CAYACSs, CACSs and YACSs, and to compare the results with normative findings; (2) To compare the rates of high neuroticism in CACSs and YACSs; (3) To compare CAYACSs with high and low neuroticism; and (4) To identify factors significantly associated with high neuroticism among CAYACSs.

## Methods

### Patient sampling

Since 1953 the Cancer Registry of Norway (CRN) has by law systematically collected notifications on all new cancer cases in the Norwegian population. The Registry contains basic data related to initial diagnosis, disease characteristics, primary treatment, and survival status. Participants eligible for the NOR-CAYACS study were identified through the Registry. Study inclusion criteria were age ≥ 18 years at time of survey, diagnosis between 1985 and 2009, and a minimum of 5 years since the initial diagnosis of any childhood and adolescent cancers (excluding central nervous system tumors due to uncertainty about their current cognitive functioning) diagnosed at ages 0–18 years (CACSs); and a selection of cancers diagnosed at ages 19–39 years (YACSs)^13,14^.

The YACSs consisted of survivors of breast cancer (stages ≤ III), colorectal cancer, non-Hodgkin lymphoma, all leukemias, and a randomly selected subsample of malignant melanomas (960 of 2873 survivors). We did not include other common cancer groups such as Hodgkin lymphoma, testicular, and cervical cancer as they were enrolled in concurrent studies at our department at the time of study inclusion*.* A questionnaire was mailed to 5361 CAYACSs, among whom 2104 responded (39% response rate). To get a sample with high cure rate, homogenous treatment, and cancer experiences, we excluded 363 CAYACSs with recurrence, 37 with distant metastases 54 with second cancers identified before survey, and six without treatment information. Fifteen respondents with incomplete neuroticism scores were also excluded from the analyses. Thereby the study sample was 1,629 CAYACSs (481 CACSs and 1148 YACSs).

### Normative data (NORMs)

The Third Health Study of Nord-Trøndelag County of Norway (the HUNT-3 study, https://www.ntnu.edu/hunt) collected laboratory and questionnaire data during 2006–2008 from all inhabitants aged ≥ 20 years. The study had 50,807 responders (response rate 54%), among which 17,463 males and 22,495 females aged 20–79 years completed the neuroticism form. The rates of self-reported high neuroticism scores of the HUNT-3 study have been published according to sex^15^, and we used these results since we did not have access to original HUNT-3 data. The HUNT findings are considered representative of the health problems of the total adult population of Norway^16^.

### Primary outcome variable

*Neuroticism* was self-rated on an abridged version of The Eysenck Personality Questionnaire (EPQ-N) with six items concerning long-term personality characteristics^17^. Each item was rated as present (1) or absent (0). The sum score ranged from zero to six, and higher score represented more neuroticism. The distribution of the sum scores were positively skewed, and we therefore applied the established dichotomization of the sum-score into the high (sum-score 3–6), and low neuroticism (sum-score 0–2) groups^18^. Internal consistency expressed as Cronbach’s coefficient alpha was 0.77 in CAYACSs and 0.73 in NORMs.

### Scales

*The Patient Health Questionnaire-9 (PHQ-9) *The PHQ-9 covered depression symptoms experienced during the last 2 weeks, and each item was scored from 0 (‘not at all’) to 3 (‘nearly every day’), providing a 0–27 severity score. A case of probable major depressive episode (MDE) was defined by a sum score ≥ 10^19,20^. Alpha was 0.88.

*The Fatigue Questionnaire (FQ) *The FQ contained mental (four items) and physical fatigue (seven items) sub-scales covering the last 4 weeks. Each item was rated from 0 (‘less than before/not at all') to 3 (‘much more than usual'). To identify cases with chronic fatigue (≥ 6 months), we used the published algorithm^21,22^. Alpha for total fatigue was 0.91.

*The Hospital Anxiety and Depression Scale (HADS) *The HADS comprised 7 items each on the anxiety and depression sub-scales rated for the last week. The item scores ranged from 0 (‘not present') to 3 (‘highly present'), so the sub-scale scores ranged from 0 (low) to 21 (high). Only the anxiety subscale was used in this study, and cases of probable anxiety disorder had a sum score ≥ 8^23^. Alpha was 0.83.

### Other variables

*Late adverse effects* (LAEs) were self-reported based on the respondents’ personal experience. Based on the literature^24–26^, 18 LAEs were listed, but we only included 14 of them which were not covered by our scales or comorbidity measure: hormonal changes, reduced fertility, dental health problems, cognitive problems, hearing problems, muscular cramps, nerve pains, numbness in hands/feet, second cancer, sexual problems, osteoporosis, lymphedema, radiation injuries, and other problems (to be specified). Only the statement of “I have personal experience” for each LAE was considered as a positive response. The number of reported LAEs was categorized into zero (reference), 1–2 LAEs, and ≥ 3 LAEs.

Self-reported *somatic diseases* were cardiovascular diseases, chronic pulmonary diseases, diabetes, kidney diseases, gastrointestinal diseases, rheumatic diseases, arthrosis, stroke, and thyroid diseases with metabolic consequences. *Comorbidity* was described as zero (reference), 1–2, and ≥ 3 reported diseases. Some of these diseases could also be LAEs, but due to lack of data concerning their relation to the malignancies and their treatments, they were classified as diseases rather than LAEs.

Information on each CAYACS’ *initial cancer type, stage, and metastases* was retrieved from the CRN, while data on cancer treatment and recurrence was self-reported. We defined four *treatment modalities:* limited surgery only (reference, as for localized melanomas), extensive local treatment (surgery and/or radiotherapy), systemic treatment only (chemotherapy and/or hormone therapy), and systemic treatment with surgery and/or radiotherapy.

*Current paired relation* was categorized as present (reference) or absent. *Level of education* was dichotomized into short (≤ 12 years) and long (> 12 years, reference). Current *work status* had six alternatives, with responses dichotomized into “in paid work” (full- or part-time work or on sick leave) (reference) versus “not in paid work” (work assessment allowance, disability pension, students, or homemakers).

*Self-rated health* had five alternatives which were dichotomized into “good health” (excellent, very good, good) (reference) versus “poor health” (moderately good, poor). *Obesity* was defined by self-reported body mass index ≥ 30, and *smoking* concerned current daily smoking of any number of cigarettes, at survey.

### Data analyses

Between-group comparisons of continuous variables were performed with independent sample t-tests. If continuous variables had skewed distribution, they were converted to categorical ones Between-group comparisons of categorical variables were performed with chi-square statistics. Since we observed significant differences between CACSs and YACSs concerning sex, all between-group analyses were adjusted for these variables using multivariable linear or logistic regression analyses with high and low neuroticism as dependent variable in Table 2. Significant differences in 10 years age groups between CACSs and YACSs were inherent in their definitions and not adjusted for.

The internal consistencies of scales were examined with Cronbach’s coefficient alpha. Independent variables were assessed in univariate and multivariable logistic regression analyses with high neuroticism as dependent variable (low neuroticism as reference). The strength of associations was expressed as odds ratios (ORs) with 95% confidence intervals (95% CI). Since the EPQ-N scale and the HADS-Anxiety subscale showed a bivariate correlation of Spearman’s rho of 0.67, the HADS-Anxiety was not included in the multivariable logistic analysis.

The p-value was set as < 0.01, and all tests were two-sided. The software applied was IBM SPSS Statistics version 26 for PC (IBM Corporation, Armonk, New York, USA).

### Ethical approval and consent to participate

The current study was approved by the Norwegian Data Protection Authority (#15/00395-2/cgn), the South-East Norway Regional Committee for Medical and Health Research Ethics (# 2015/232 REK Sør-Øst B), The Data Protection Officer at Oslo University, and the CRN approved the NOR-CAYACS study. All participants signed an informed consent form. The study was conducted in accordance with relevant guidelines and regulations, and the Declaration of Helsinki.

## Results

### Responders versus non-responders

Among CAYACSs we had data on sex and age of both responders and non-responders. Among responders, females were significantly over-represented, as were respondents > 40 years. The younger age groups were under-represented among respondents. The mean age was significantly higher among respondents compared to non-respondents (data not shown).

### Description of the CAYACSs

Median age at first cancer diagnosis was 31 years (range 0–39 years), median age at survey was 45 years (range 18–64 years), and median time from first diagnosis to survey 16 years (range 5–30 years). Findings concerning cancer types, treatment groups, and LAEs are displayed in Table 1.

**Table 1:** Characteristics of childhood and adolescent (CACSs), young adult (YACSs) and all cancer survivors at survey.

Variables	CACSs (N = 481)	YACSs (N = 1148)	p-value	CAYACS (N = 1629)
**Age at first diagnosis (years)**			–	
Median (range)	12 (0–18)	34 (19–39)	NA	31 (0–39)
Age at survey, median (range)	29 (18–49)	48 (26–64)	NA	45 (18–64)
Time since first diagnosis, median (range)	19 (5–30)	14 (5–30)	**< 0.001**	16 (5–30)
**Age groups at survey, N (%)**			**< 0.001**	
18–29 years	253 (53)	9 (1)		262 (16)
30–39 years	152 (31)	119 (10)		271 (17)
40–49 years	76 (16)	537 (47)		613 (38)
50–59 years	0 (0)	362 (32)		363 (22)
60–64 years	0 (0)	120 (10)		120 (7)
**Sex, N (%)**			**< 0.001**	
Males	201 (42)	303 (26)		504 (31)
Females	280 (58)	845 (74)		1125 (69**)**
**Types of cancer, N (%)**	–		NA	–
Melanomas	–	272 (24)		272 (17)
Breast	–	453 (40)		453 (28)
Colo-rectal	–	126 (11)		126 (8)
Non-Hodgkin lymphomas	–	177 (15)		177 (11)
Leukemias	–	120 (10)		120 (7)
Leukemias	162 (34)	–		162 (10)
Lymphomas	127 (26)	–		127 (7)
Solid tumors	192 (40)	–		192 (12)
**Treatment groups, N (%)**	–	–	**< 0.001**	–
Limited surgery only	54 (11)	369 (32)	**< 0.001**	423 (26)
Local treatment	29 (6)	59 (5)	0.91	88 (5)
Systemic treatment only	173 (36)	185 (16)	0.018	358 (22)
Systemic + surgery/radiation	225 (47)	535 (47)	0.21	760 (47)
**Late adverse effects, N (%)**			**< 0.001**	
None	217 (45)	489 (43)	0.36	706 (43)
1–2	164 (34)	287 (25)	**0.002**	451 (28)
3+	99 (21)	368 (32)	**< 0.001**	467 (29)
**Neuroticism, N (%)**			**< 0.001**	
High	211 (44)	397 (35)		608 (37)
Low	270 (56)	751 (65)		1021 (63)

Significant values are in bold.

Sixty-nine percent of the CAYACSs were females, and 74% lived in paired relationships. Short education was reported by 57% of the sample, and 75% were in paid work. Eighty percent reported good health, and 63% reported ≥ 1 comorbid disease. Between 18 and 22% reported case levels of anxiety or depression, or chronic fatigue. Fourteen percent of the CAYACSs were obese, and 10% daily smokers (Table 1).

### Comparisons of CACSs and YACSs

Median age of CACSs at diagnosis and at survey was lower than for YACSs, and they had significantly longer median follow-up time (Table 1). Significantly fewer CACSs than YACSs had limited surgery only and significantly more had received systemic treatment. CACSs had significantly more of 1–2 LAEs, and significantly less of ≥ 3 LAEs compared to YACSs (Table 1).

### Rates of high neuroticism

The rate of high neuroticism in the CAYACSs was 37% (95% CI 35–40%), 30% (95% CI 26–34%) among males and 40% (95% CI 38–43%) among females (p < 0.001). Similar significant sex differences were also found for the CACSs (34% versus 51%) and YACSs (27% versus 37%) subgroups. Female CACSs had significantly increased rate of high neuroticism (51%) compared to female YACSs (37%), while the difference in rates was non-significant among male CACSs and YACSs (34% vs 27%). However, the total CACSs had significantly higher occurrence of high neuroticism than the total YACSs group (44% versus 34%) (Table 2).

**Table 2:** Number and percent with low and high neuroticism among CAYACS versus NORMs and CACSs versus YACSs according to sex.

Neuroticism sum scores	CAYACS	NORMs	p-value	CACSs	YACSs	p-value
	N (%)	N (%)		N (%)	N (%)	
**Males**						
Low	352 (70)	13,902 (80)	**< 0.001**	132 (66)	220 (73)	0.08
High	152 (30)	3561 (20)		69 (34)^b^	83 (27)^c^	
Sum	504 (100)	17,463 (100)	NA^a^	201 (100)	303 (100)	NA
**Females**						
Low	669 (60)	15,030 (67)	**< 0.001**	138 (49)	531 (63)	**< 0.001**
High	456 (40)	7465 (33)		142 (51)^b^	314 (37)^c^	
Sum	1125 (100)	22,495 (100)	NA	280 (100)	845 (100)	NA
**Total samples**						
Low	1021 (63)	28,932 (72)	**< 0.001**	270 (56)	751 (66)	**< 0.001**
High	608 (37)	11,026 (28)		211 (44)^b^	397 (34)^c^	
Sum	1629 (100)	39,958 (100)	NA	481	1148	NA

Significant values are in bold. ^a^NA: Non-applicable ^b^CACs versus NORMs p < 0.001; ^c^YACSs versus NORMs p ≤ 0.025

Compared to NORMs, the occurrence of high neuroticism was significantly higher for total CAYACSs (37% vs 28%) and for both sexes (males 30% vs 20%, females 40% vs 33%). For males, females, and total samples both CACSs and YACSs had significantly higher occurrences of high neuroticism than NORMs (Table 2).

### Comparison of high versus low neuroticism groups

The high neuroticism group had significantly lower proportions of survivors in paired relations, with long education, and in paid work. The high neuroticism group also had a significantly higher percentages reporting LAEs, poor self-rated health and comorbid diseases. Further, the proportions of survivors with chronic fatigue, cases of anxiety and depression, obesity, and daily smoking were all significantly increased in the high versus low neuroticism group. (Table 3).

**Table 3:** Findings in the high and low neuroticism groups at survey.

Variables	High neuroticism (N = 608)	Low neuroticism (N = 1021)	p-value	Total sample (N = 1629)
**Sex, N (%)**			**< 0.001**	
Males	152 (25)	352 (34)		504 (31)
Females	456 (75)	669 (66)		1125 (69)
Age at survey. years, mean (SD)	41.2 (11.8)	44.3 (11.2)	**< 0.001**	43.1 (11.5)
**Age groups at survey, N (%)**			**< 0.001**	
18–29 years	125 (21)	137 (13)		262 (16)
30–39 years	116 (19)	155 (15)		271 (17)
40–49 years	220 (36)	393 (39)		613 (38)
50–59 years	112 (18)	251 (25)		363 (22)
60–64 years	35 (6)	85 (8)		120 (7)
**CAYACS, N (%)**			< 0.001*	
CACSs	211 (44)	397 (35)		608 (37)
YACSs	270 (56)	751 (65)		1021 (63)
**Late adverse effects, N (%)**			**< 0.001**	
None	196 (32)	510 (50)		706 (43)
1–2	180 (30)	271 (27)		451 (28)
3+	229 (38)	238 (23)		467 (29)
In paired relationship, N (%)	422 (68)	797 (78)	< 0.001*	1209 (74)
**Level of basic education, N (%)**			0.001*	
Long (> 12 years)	324 (52)	614 (60)		928 (57)
Short (≤ 12 years)	291 (48)	402 (40)		693 (43)
**Work status, N (%)**			< 0.001*	
In paid work	385 (65)	813 (81)		1198 (75)
Not in paid work	208 (35)	186 (19)		394 (25)
**Comorbidities, N (%)**			< 0.001*	
None	178 (29)	430 (42)	< 0.001*	608 (37)
1–2	341 (56)	513 (50)	< 0.001*	854 (53)
3+	89 (15)	78 (8)	< 0.001*	167 (10)
**Self-rated health, N (%)**			< 0.001*	
Good	382 (63)	911 (89)		1293 (80)
Poor	221 (37)	108 (11)		329 (20)
Chronic fatigue cases, N (%)	248 (41)	103 (10)	< 0.001*	351 (22)
Anxiety cases, N (%)	297 (49)	49 (5)	< 0.001*	346 (21)
Depression cases, N (%)	244 (40)	44 (4)	< 0.001*	288 (18)
Obesity (BMI ≥ 30), N (%)	103 (17)	126 (12)	0.008*	229 (14)
Daily smoking, N (%)	81 (13)	82 (8)	0.002*	163 (10)

Significant values are in bold.

*Adjusted for sex.

### Bivariate and multivariable analyses

In bivariate analyses younger age at survey, years since diagnosis, CACSs versus YACSs, more intense treatment, increasing number of LAEs and comorbidities, poor self-rated health, being female, short education, not being in paired relationship or in paid work, having chronic fatigue, being cases of anxiety or depression, or being obese or daily smoker were all significantly associated with high neuroticism (Table 4).

**Table 4:** Bivariate and multivariable logistic regression analyses of independent variables and high neuroticism (N = 608) as dependent variable [low neuroticism (N = 1021) as reference] in CAYACSs (N = 1629).

Variables	Bivariate analyses			Multivariable analysis		
	OR	95% CI	p-value	OR	95% CI	p-value
Age at survey	0.98	0.97–0.99	**< 0.001**	0.97	0.95–0.99	**0.019**
Time since diagnosis	0.98	0.97–1.00	0.034	1.00	0.98–1.03	0.83
CACSs (YACSs reference)	1.48	1.19–1.84	**< 0.001**	1.00	0.56–1.74	0.99
**Treatment groups**			**0.001**			0.39
Surgery only (reference)	1.00	–	**–**	1.00	–	**–**
Local treatment	1.39	0.86–2.26	0.18	0.74	0.40–1.38	0.34
Systemic treatment only	1.68	1.25–2.26	**0.001**	0.73	0.48–1.11	0.14
Systemic + others	1.65	1.28–2.13	**< 0.001**	0.73	0.50–1.07	0.11
**Late adverse effects**			**< 0.001**		–	**0.038**
None (reference)	1.00	–	**–**	–	–	**–**
1–2	1.73	1.35–2.22	**< 0.001**	1.32	0.96–1.67	0.11
3+	2.50	1.96–3.20	**< 0.001**	1.62	1.12–2.34	**0.011**
**Comorbidities**			**< 0.001**			0.25
None (reference)	1.00	–	**< 0.001**	–	–	**–**
1–2 disease(s)	1.61	1.29–2.01	**< 0.001**	1.27	0.96–1.67	0.10
3 + diseases	2.76	1.94–3.91		1.20	0.74–1.94	0.46
Poor self-rated health	4.88	3.77–6.32	**< 0.001**	1.61	1.14–2.29	**0.008**
Female (male reference)	1.58	1.26–1.98	**< 0.001**	1.77	1.32–2.36	**< 0.001**
Non-paired relationship	1.69	1.35–2.12	**< 0.001**	1.21	0.91–1.63	0.19
Short education	1.42	1.16–1.73	**< 0.001**	1.22	0.94–1.58	0.13
Not in paid work	2.36	1.87–2.98	**< 0.001**	0.90	0.65–1.24	0.51
Chronic fatigue case	6.14	4.73–7.96	**< 0.001**	1.85	1.38–2.47	**< 0.001**
Anxiety case	18.95	13.65–26.31	**< 0.001**	MC		
Depression case	14.98	10.63–21.11	**< 0.001**	1.75	3.57–1.95	**< 0.001**
Obesity	1.46	1.10–1.94	**< 0.001**	1.00	0.56–1.76	0.99
Daily smoking	1.76	1.27–2.44	**< 0.001**	1.21	0.80–1.82	0.37

Significant values are in bold.

*MC* multicollinearity between anxiety and neuroticism (Spearman’s rho 0.67).

In the multivariate analysis younger age at survey, ≥ 3 LAEs, poor self-rated health, being female, having chronic fatigue, and having depression remained significantly associated with high neuroticism.

## Discussion

In CAYACSs the rate of high neuroticism was significantly increased compared to NORMs both in the total sample as well as for male and female CAYACSs. In both CACSs and YACSs the occurrence of high neuroticism was significantly higher than in NORMs. High neuroticism was significantly more common in female CACSs versus female YACSs, while that difference was insignificant between male CACSs and male YACSs.

In the bivariate analyses both cancer-related, sociodemographic, comorbidities and poor health, anxiety and depression, chronic fatigue, obesity, and daily smoking were significantly associated with high neuroticism. In the multivariable analysis younger age at survey, ≥ 3 LAEs, poor health, being female, cases of chronic fatigue, and cases of depression remained significantly associated with high neuroticism.

In population-based studies the rates of high neuroticism are increased in females compared to males and in younger adults compared to older ones^2,5^. In this perspective corresponding findings among CAYACSs could just confirm general sex and age issues. However, since all groups of survivors showed significantly increased rates of high neuroticism compared to NORMs, we may hypothesize that the cancer experience could increase the risk of high neuroticism in young cancer survivors. This finding is in line with previous findings in short-term survivors of childhood cancer^10^. Our interpretation is that the experience of cancer between birth and the age of 39 years, increases the risk of high neuroticism in CAYACSs compared to NORMs. This hypothesis is in line with studies reporting increase in neuroticism in persons exposed to life-threatening traumas during childhood and adolescence^27,28^ although such findings are still methodologically controversial according to recent reviews^2,3^.

By our definition more than one third (37%) of CAYACSs have the vulnerability factor of high neuroticism. In the general population such vulnerability implies increased risk for somatic diseases, particularly cardiovascular ones^5^, mental disorders^4^, unhealthy lifestyle^6^, dementia^29^, and suicide^30^. High neuroticism may thus considerably increase the illness burden of being CAYACSs. Our results corroborate the strong associations between high neuroticism and many aspects of the somatic and mental health of long-term CAYACSs. In general, the influence of high neuroticism on healthcare is considerable^5^, and so is also its cost influence on health care^31^. The increased rate of high neuroticism in CAYACSs should have considerable implications for their follow-up care, and, therefore, should be in the mind of their health care providers. High neuroticism may be considered as a causal factor associated with long-term outcomes, eventually mediated through unhealthy lifestyles manifested by obesity, daily smoking, or minimal physical activity^5^. If counselling by health care providers about a healthier lifestyle shall be effective, high neuroticism must be considered^32^.

Individuals with high neuroticism more easily and more strongly experience negative emotional reactions to stress. High neuroticism is the backbone of anxiety, depression, and mental trauma symptoms^4,5^. In the clinic CAYACSs with high neuroticism are likely to need more explanations, encouragement, and reassurance from health care professionals than CAYACSs with lower levels of neuroticism.

Personality traits like neuroticism are typically defined as characteristic and automatic patterns of thinking feeling and behaving that are consistent over time and across situations^2^. Deviations in personality traits should not be considered as illnesses, but as risk factors, and high neuroticism is a risk factor for somatic and mental health problems as well as unhealthy lifestyle^4–6^. However, high neuroticism also is health protective, especially when combined with high conscientiousness (“healthy neuroticism”)^9^. Health care professionals could easily develop negative value judgments concerning the multiple complains of patients of patients with high neuroticism forgetting that they need of more comprehensive care than patients with low neuroticism. Professionals should also be aware that high neuroticism may be modified by a variety of psychological and pharmacological interventions^33^.

Due to their stability over time personality traits like neuroticism are considered as traits, while other variables like anxiety or depression, are classified as states being more short-lived and instable over time. The stability of personality traits is established before young adulthood, and they show only minor changes from that time and until the survey where the CAYCSs have a median age of 45 years. The timeline and the trait-state dichotomy are both important for the interpretation of our cross-sectional study. However, it should be noted that the stability versus plasticity of basic personality trait currently is a matter of considerable controversy among researchers in the field^2,3^.

A major strength of our study is the large population-based sample size and inclusion of a wide range of variables potentially associated with neuroticism. A further strength is our use of self-rating instruments with established psychometric properties. Although we could not match our NORMs with the CAYACSs on age and sex, we still consider their comparisons a strength of our study.A limitation is the response rate of 39% and age- and sex biases among the CAYACSs responding. The response rate is in line with national and international trends concerning questionnaire studies^34,35^. The attrition analysis showed that the responding CAYACSs are somewhat biased compared to the whole sample invited to the study. Our study did not include all types of cancer occurring during childhood, adolescence, and young adulthood. Further, considering the length of our questionnaire, we did not include the other “Big five” personality traits, thereby missing interesting data. Based on other studies we choose the sum score of ≥ 3 for our definition of high neuroticism, Another cut-off could eventually imply different results. Another limitation is the lack of a trauma instrument in our questionnaire. We thereby missed the CAYACs’ opinions of their cancer experiences as traumas eventually associated with development of high neuroticism. Our findings must be interpreted with these limitations of representativity in mind.

## Conclusion

CAYACSs, CACSs, and YACSs all have increased rates of high neuroticism compared to NORMs. Female CACSs have significantly increased rate of high neuroticism than female YACSs suggesting that earlier cancer trauma in females increases the risk of high neuroticism. High neuroticism is significantly associated with many negative health outcomes in CAYACSs. Identification of high neuroticism using a simple screening test should be considered by healthcare providers caring for CAYACSs. Such identification is important for the consultation approach to CAYACSs and to management of their many health risks. Due to its multiple associations with important health outcomes, high neuroticism should become a focus of cancer survivorship.

## Supplementary Information

Supplementary Information.

## Data Availability

The dataset used for analyses of the study resides with the senior author Hanne C. Lie.

## Abbreviations

95% CI: 95% Confidence interval
CACSs: Childhood and adolescence cancer survivors
CAYACSs: Childhood, adolescence, and young adult cancer survivors
CRN: Cancer Registry of Norway
EPQ-N: Eysenck Personality Questionnaire-Neuroticism
FQ: The Fatigue Questionnaire
HADS: The Hospital Anxiety and Depression Scale
HUNT-3: The Third Health Study of Nord-Trøndelag County of Norway
LAEs: Late adverse effects
NORMs: Normative data sample
PHQ-9: The Patient Health Questionnaire 9
YACSs: Young adult cancer survivors
