# Supplementary material for: A study of high neuroticism in long-term survivors of childhood, adolescence, and young adult cancers
Source: Sci Rep. 2022 Jul 19;12:12325. doi: 10.1038/s41598-022-15697-3 (PMC9296654; doi:10.1038/s41598-022-15697-3)
Supplement: Supplementary file 1 — Supplementary Information. [file 41598_2022_15697_MOESM1_ESM.docx]

**Supplement.**

**The 6-item version of neuroticism based on the Eysenck Personality Questionnaire.**

**Instruction:** The items below concern how you usually behave, feel, or act. Please, set a ring round the number for either Yes or No for each item. Please, respond quickly and do not think too long about the meaning of each item.

| **Items** | **Yes** | **No** |
| --- | --- | --- |
| 1. Are you often worried? | 1 | 0 |
| 1. Are your feelings easily hurt? | 1 | 0 |
| 1. Do you often feel that you lose interest? | 1 | 0 |
| 1. Do you have nervous problems? | 1 | 0 |
| 1. Do you often feel tired and indifferent/unmotivated without reason? | 1 | 0 |
| 1. Do you worry that terrible things might happen? | 1 | 0 |

Low neuroticism score: sum score 0 – 2; High neuroticism score: sum score 3 – 6) (reference #14).
